# Acute lung injury and the role of histones

**DOI:** 10.1186/2213-0802-2-1

**Published:** 2014-01-03

**Authors:** Peter A Ward, Jamison J Grailer

**Affiliations:** Department of Pathology, University of Michigan Medical School, 1301 Catherine Rd, Box 5602, Ann Arbor, MI 48109 USA

**Keywords:** C5a, Histones, ALI, C5a receptors

## Abstract

Acute respiratory distress syndrome (ARDS) in humans involves ≥ 200,000 individuals in the United States, and has a mortality rate (40%) for which no specific drug has been approved for use in humans. We have studied experimental acute lung injury (ALI) in mice following airway deposition of bacterial lipopolysaccharide (LPS) or the recombinant mouse complement anaphylatoxin, C5a. As ALI developed over 6 hr, extracellular histones appeared in bronchoalveolar lavage fluids (BALF). Extracellular histone appearance required both C5a receptors (C5aR, C5L2) as well as neutrophils (PMNs) and lung macrophages, as genetic loss of either C5a receptor or depletion of PMNs or macrophages reduced histone levels found in BALF during ALI. It is possible that extracellular histones were derived from formation of neutrophil extracellular traps (NETs) in lung after PMN contact with C5a. When purified histones were delivered to lung via the airways, intense inflammatory injury ensued and type II cells developed large blebs indicating cellular damage and apoptosis. Detailed physiological measurements revealed severe disruption of blood/alveolar gas exchange. These data suggest a key role for histones in development of experimental ALI.

## Introduction

Extracellular histones have recently been recognized as appearing in plasma after endotoxemia or septic shock in rodents and in non-human primates with septic shock following infusion of live *E. Coli* bacteria [1]. In mice, infusion of a histone mixture (purified from calf thymus) led to rapid death which was likely linked to histone binding to vascular endothelial cells resulting in endothelial cell apoptosis [1]. Histones have also been implicated in experimental acute liver injury (ischemia/reperfusion) and experimental kidney injury (endotoxin-induced) [2, 3]. In both cases, use of a neutralizing antibody to histones was highly protective. In these examples of “sterile inflammation”, there was evidence that Toll-like receptor (TLR) 2 and TLR4 were involved in the development of acute kidney injury, and TLR9 was linked to liver injury, perhaps functioning as receptors for histone binding to cells [2, 3]. There is also evidence in human acute respiratory distress syndrome (ARDS) that histones were present in bronchoalveolar lavage fluids (BALF) [4]. Plasma histones have recently been described in patients who developed ARDS after non-penetrating polytrauma [5]. On a cellular level, extracellular histones are known to act as damage-associated molecular patterns (DAMPs) that can activate TLRs resulting in pro-inflammatory cytokine production [2, 3]. In addition, high levels of extracellular histones are cytotoxic to endothelial and epithelial cells [1, 4, 5], as well as several other cell types (JJG and PAW, unpublished observations). The mechanism of the cytotoxic effects of extracellular histones is not entirely clear, but appear to be due to their ability to interact with phospholipids in the plasma membrane leading to increased transmembrane conductance, calcium influx, cell swelling and cytolysis [5, 6]. In vivo, the cytotoxic effects of histones may result in the further release of DAMPs leading to enhanced inflammation.

One possible source of histones is neutrophils (PMNs) that both in vitro and in vivo can react with C5a to form neutrophil extracellular traps (NETs) composed of DNA and histones, as well as products from PMN granules such as myeloperoxidase, which collectively trap bacteria, leading to their killing [7–9]. In the following report, we will review our evidence in experimental acute lung injury (ALI) that histones appear in BALF in a complement and C5a receptor-dependent manner. Evidence will be provided to directly link histone presence with development of ALI.

## Review

### Induction of ALI and requirements for C5a receptors and PMNs

All rodent procedures were performed within the U.S. National Institutes of Health guidelines and were approved by the University of Michigan Committee on the Use and Care of Animals. Airway instillation of 1 μg LPS or C5a into mouse lung produced an accumulation of mouse albumin in BALF, assessed by ELISA, that was nearly 10 fold greater than in negative control lungs from mice given sterile saline intratracheally (i.t.) [4]. Albumin leakage into lung is the result of both non-specific epithelial/endothelial damage as well as active modification of tight junctions [10, 11]. As would be expected, large accumulations of PMNs in BALF occurred in the positive controls (3–5 × 10^6^). When C5a receptor knockout mice were used (C5aR^-/-^, C5L2^-/-^), there was greatly reduced albumin leak into lung (75-90%) and PMN counts in BALF were very much reduced (60-80%). A key accompanying finding was that in either C5aR^-/-^ or C5L2^-/-^ mice, histone levels were reduced ≥ 90%, based on ELISA, indicating that histone appearance was C5a receptor-dependent. It was also demonstrated in wild type (Wt) mice depleted of PMNs with anti-Ly6G (neutrophil-specific epitope) antibody that there was a 70% reduction of histones in BALF.

### Requirements for histones in development of ALI

In the C5a model of ALI, a neutralizing mAb to H4 histone was employed in mice. 250 μg was given i.v. and 50 μg i.t., resulting in the leak of albumin being reduced by nearly 50% in BALF, and there was a broad reduction in BALF levels of proinflammatory cytokines and chemokines [4]. These data indicated that histones play an important role in development of the leak of albumin into lung as well as the appearance of a broad spectrum of proinflammatory cytokines and chemokines in BALF.

### Ability of preformed histones to cause acute injury to lung

A mixture of histones (20 μg/gm body weight) was injected i.t. into Wt mouse lungs, resulting in lung consolidation (as defined by high resolution MRI), consistent with intense accumulation of water and PMNs [4]. Hematoxylin and eosin staining of paraffin-embedded lung tissue revealed intense alveolar edema and buildup of PMNs, together with sloughing of airway epithelial cells and development of venous thrombi. Electron microscopic images revealed significant blebbing of alveolar epithelial cells, indicating cell damage and/or apotosis. Functional measurements showed arterial acidosis, elevations in arterial pCO_2_, and arterial pO_2_ desaturation, together with a greatly amplified respiratory rate, increased minute ventilation time and a greatly reduced total respiratory cycle. All of this indicates severe functional disturbances in lungs of mice receiving histones via the airways.

### The source of extracellular histones during ALI

The cellular source of extracellular histones remains unclear. Histones are reported to be released from apoptotic/necrotic cells in vitro [3, 12]. Therefore, histones may be released by damaged cells during ALI. In addition to this possibility, histones are known to be associated with neutrophil extracellular traps (NETs) [13]. NETs are structures composed of DNA, histones, and granular proteins (e.g., elastase, myeloperoxidase) released by neutrophils in response to a variety of agonists. NET release has been demonstrated during experimental ALI [9]. Therefore, histones in the extracellular space may be from dead/dying cells, from neutrophils, or from both sources. Further investigation is needed to clarify the source of extracellular histones during ALI.

### Evidence for extracellular histones during ARDS

The presence of extracellular histones in BALF from patients with ARDS was confirmed by both Western blot and ELISA technology. Extracellular histones were detected in 50% of patient BALFs from 0–10 days after initial diagnosis (n = 28). Lower rates of histone presence was observed in BALF samples collected >10 days after diagnosis (n = 24). There were no detectable histones in BALFs from healthy control samples (n = 12). In serial samples from single patients (e.g., collected 5, 14, and 21 days after initial diagnosis), histone presence was sustained in some (observed in BALF samples from multiple days). In other patients, histones were only present in early samples. Information about the patient sampling for this study is described elsewhere [14]. The original human study was approved by the Institutional Review Board of the University of Michigan Health System.

## Conclusions

**Figure 1:**
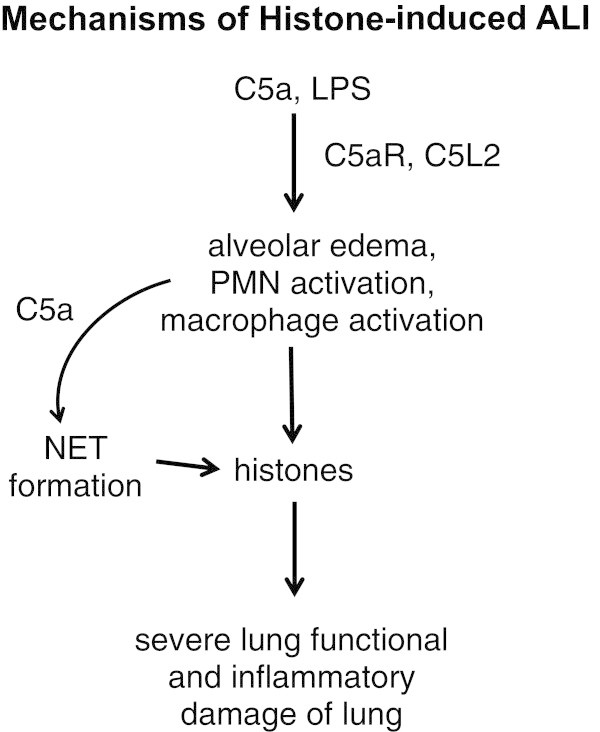
**Mechanisms of histone-induced ALI.**

Figure 1 outlines our current thoughts on the development of ALI following airway deposition of C5a or LPS. In each case, both C5a receptors were vital to development of lung injury. Absence of either C5aR or C5L2 greatly attenuated ALI as quantitated by leak of albumin into lung, buildup of PMNs and appearance of proinflammatory cytokines and chemokines in BALF. It has already been shown by others that PMN contact with C5a can cause NET formation [8, 9], with the appearance of histones and other bactericidal products from granules of PMNs. There may well be other cell sources of histones in the lung. Extracellular histones, once formed, had intense lung damaging effects that markedly interfered with air gas exchange between the capillary and alveolar compartments. Accordingly, it is possible in ARDS that histones may represent a target for blockade. One such candidate, activated protein C (APC), has been shown to hydrolyze histones [1]. Inhaled APC was beneficial during rodent and sheep experimental ALI [15, 16]. However, a phase II clinical trial which administered APC i.v. in patients with ARDS showed no improvement in outcome [17]. The efficacy of inhaled APC has not been determined in humans with ARDS, but might represent a promising therapy [18]. Another potential therapeutic for ARDS may be the development of humanized blocking/neutralizing antibodies for histones.

## Abbreviations

ALI: Acute lung injury
ARDS: Acute respiratory distress syndrome
APC: Activated protein C
BALF: Bronchoalveolar lavage fluid
ELISA: Enzyme-linked immunosorbent assay
H4: Histone H4
LPS: Lipopolysaccharide
mAb: Monoclonal antibody
NETs: Neutrophil extracellular traps
PMNs: Neutrophils
TLR: Toll-like receptor
Wt: Wild type.
